# Differentiation of intraductal papillary mucinous neoplasms and pancreatic ductal adenocarcinoma using arterial-phase CT radiomics combined with clinical features

**DOI:** 10.3389/fonc.2026.1835753

**Published:** 2026-07-17

**Authors:** Min Feng, Shicheng Feng, Zhiqiang Lu, Miao Lu, Jie Shen

**Affiliations:** 1 Radiotherapy Department, Zhongda Hospital, Southeast University, Nanjing, Jiangsu, China; 2 Hepatobiliary Surgery Department, Zhongda Hospital, Southeast University, Nanjing, Jiangsu, China; 3 Radiology Department, The Affiliated Brain Hospital of Nanjing Medical University, Nanjing, Jiangsu, China

**Keywords:** cancer, computed tomography, intraductal papillary mucinous neoplasm, pancreatic ductal adenocarcinoma, radiomics

## Abstract

**Objective:**

To investigate the value of arterial-phase CT-based radiomics combined with clinical features in differentiating IPMN (Intraductal Papillary Mucinous Neoplasms) from PDAC (Pancreatic Ductal Adenocarcinoma).

**Methods:**

A total of 216 patients with pathologically confirmed IPMN or PDAC were retrospectively enrolled. Clinical data and contrast-enhanced CT images were collected. Patients were divided into a training cohort, a test cohort and the external validation cohort. Univariate and multivariate analyses were performed on clinical variables and CT features to identify independent predictors. Regions of interest (ROIs) were manually delineated using ITK-SNAP software, and radiomics features were extracted with the Pyradiomics package. Feature dimensionality reduction and selection were conducted using the least absolute shrinkage and selection operator (LASSO) method. A radiomics score was calculated, and radiomics and combined models were constructed using a random forest (RF) algorithm. The diagnostic performance and clinical utility of each model were evaluated.

**Results:**

Multivariate analysis identified single cystic lesion (OR = 2.33), mural nodules (OR = 2.69), and CA19–9 level (OR = 2.36) as independent factors for differentiating IPMN from PDAC (all *P* < 0.05). Radiomics features extracted from arterial-phase contrast-enhanced CT images were used to construct a radiomics model. A combined model incorporating both radiomics and clinical features demonstrated superior predictive performance. The area under the receiver operating characteristic curve (AUC) of the combined model was 0.935 (95% CI: 0.898-0.974) in the training cohort and 0.931 (95% CI: 0.846-1.000) in the test cohort, which was significantly higher than that of the radiomics model alone (AUC = 0.885 and 0.827, respectively). The AUC of the external validation cohort was 0.808 (95% CI:0.684-0.968), and calibration curves showed good agreement between predicted and observed outcomes. Decision curve analysis indicated that the combined model provided greater clinical benefit.

**Conclusion:**

A predictive model based on arterial-phase CT radiomics combined with clinical features can effectively differentiate IPMN from PDAC.

## Introduction

IPMNs arise from mucin-secreting epithelial cells of the pancreatic ductal system and are classified as epithelial tumors. They exhibit a stepwise progression from low-grade dysplasia to high-grade dysplasia and may ultimately evolve into invasive adenocarcinoma; therefore, IPMNs are regarded as premalignant lesions of pancreatic cancer (1). PDAC, an aggressive malignant tumor, has an annual incidence increase of approximately 0.5%. Despite recent advances in detection and treatment, its nonspecific clinical manifestations and lack of symptoms at early stages make diagnosis challenging (2). Consequently, nearly 70% of patients with PDAC miss the opportunity for curative surgical resection, and the overall 5-year survival rate remains as low as 13% (3). Recent studies have shown that nanobiomaterial composites can improve the therapeutic efficacy for pancreatic cancer (4).

Previous studies have shown that the prognosis of IPMN is generally better than that of PDAC; however, the recurrence rate can be as high as 43% (5). Elevated carbohydrate antigen 19-9 (CA19-9) levels and lymph node enlargement have been identified as robust predictors of postoperative progression from IPMN to PDAC. Nevertheless, substantial overlap exists between IPMN and PDAC in terms of CT imaging appearances and pathological features, which may lead to misdiagnosis and unnecessary patient morbidity (6, 7).

Radiomics is a noninvasive approach that enables high-throughput extraction of a large number of quantitative features from medical images, allowing assessment of intertumoral and intratumoral heterogeneity that is not discernible by visual inspection alone (8, 9). Computed tomography (CT), which is widely available and offers high spatial and temporal resolution, is one of the most commonly used imaging modalities for the evaluation of IPMN and PDAC. Previous studies have demonstrated that CT-based radiomics can improve the differential diagnosis of IPMN (10). Accordingly, the present study aims to investigate contrast-enhanced CT-based radiomics features to provide imaging evidence for the clinical differentiation of IPMN and PDAC.

## Materials and methods

### Study population

**Figure 1:**
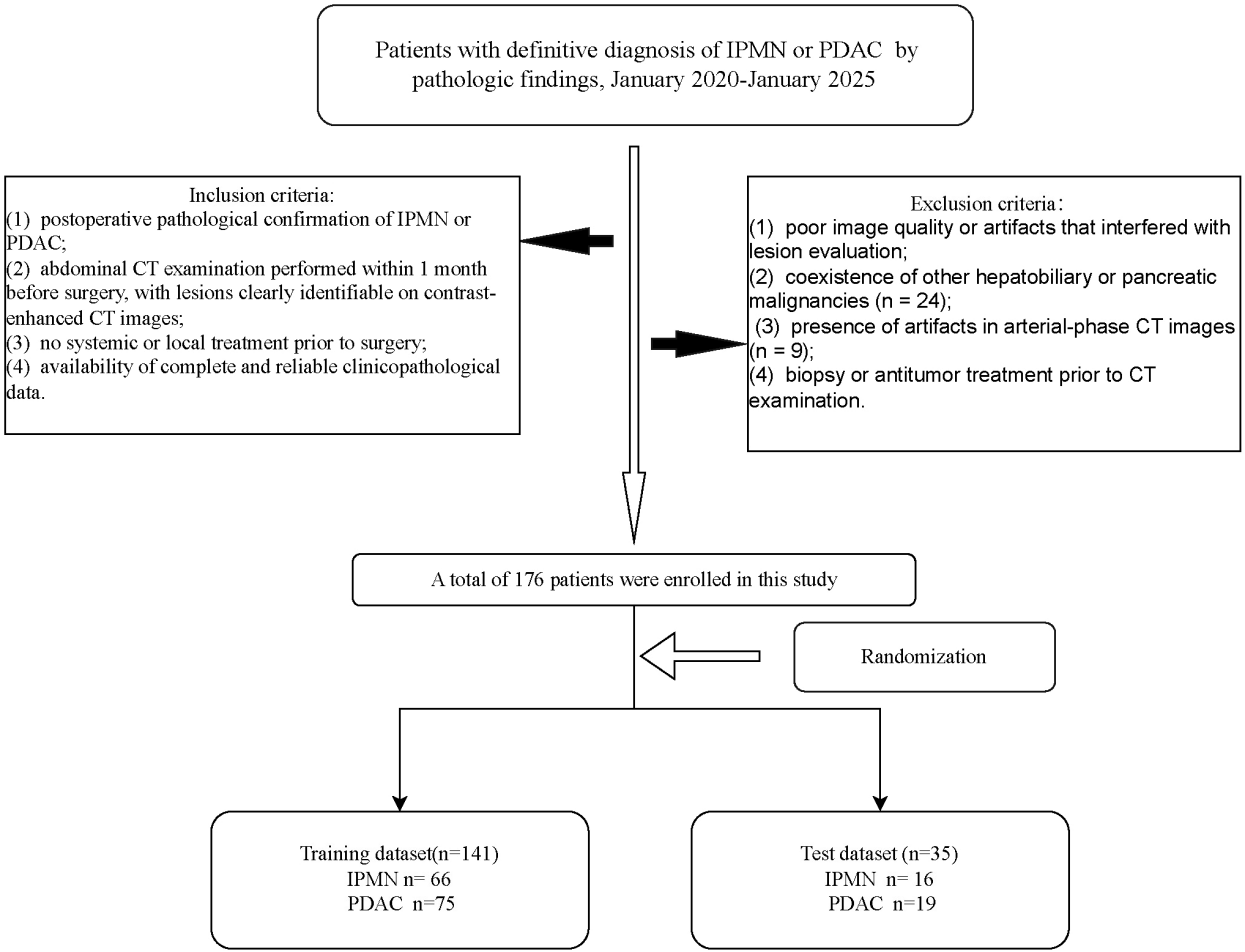
Flowchart of inclusion and exclusion criteria for the patients.

Clinical and CT imaging data of 216 patients with IPMN or PDAC treated between January 2020 and January 2025 were retrospectively analyzed (**Figure 1**). The inclusion criteria were as follows: (1) postoperative pathological confirmation of IPMN or PDAC; (2) abdominal CT examination performed within 1 month before surgery, with lesions clearly identifiable on contrast-enhanced CT images; (3) no systemic or local treatment prior to surgery; and (4) availability of complete and reliable clinicopathological data. The exclusion criteria were: (1) poor image quality or artifacts that interfered with lesion evaluation; (2) coexistence of other hepatobiliary or pancreatic malignancies (n = 24); (3) presence of artifacts in arterial-phase CT images (n = 9); and (4) biopsy or antitumor treatment prior to CT examination. All patients’ contrast-enhanced CT images and clinical characteristics were included for analysis. According to pathological diagnosis, patients were divided into the IPMN group and the PDAC group. The cohort was randomly split into a training cohort and a test cohort at a ratio of 8:2. The training cohort included 141 patients (66 IPMN and 75 PDAC), the test cohort included 35 patients (16 IPMN and 19 PDAC), and the external validation cohort (20 IPMN and 20 PDAC). All preprocessing, feature selection, and model generation procedures were conducted only on the training sample set, whereas the remaining samples were utilized solely for evaluation purposes. This study was approved by the Institutional Ethics Committee (approval number: 2025ZDSYLL060-Y01), and the requirement for informed consent was waived.

### Imaging protocol

All patients underwent contrast-enhanced abdominopelvic CT using a Revolution 256-slice CT scanner (GE HealthCare). Scanning was performed with the patient in the supine position, covering the area from the diaphragm to the level of the ischial tuberosities. The CT acquisition parameters were as follows: tube voltage, 120 kV; tube current, automatically modulated; slice thickness and interval, 5.0 mm; matrix size, 512 × 512. After unenhanced scanning, nonionic iodinated contrast material (iohexol, 1.5 mL/kg) was administered via antecubital venous injection using a bolus technique, and arterial-phase images were acquired with a delay of 30–40 s.

### Image analysis

Arterial-phase contrast-enhanced CT images in DICOM format were obtained for analysis. Two radiologists with more than 10 years of experience in diagnostic imaging independently reviewed the images in a blinded manner. The following imaging features were evaluated: lesion location (pancreatic head vs. body/tail), maximum diameter, single versus multiple cysts, presence of mural nodules, and calcifications. CT images were resampled to isotropic voxels of 1 mm³ and presented using abdominal soft-tissue window settings (width: 300 HU; level: 50 HU).

### Radiomics feature selection and model construction

DICOM images were imported into ITK-SNAP software (version 4.4.0; www.itksnap.org) for segmentation of the ROIs (11). Radiologist B reviewed the segmentation performed by radiologist A, and discrepancies were resolved by consensus. Forty samples were randomly chosen and separately allocated. The intra-group correlation coefficients for each characteristic were computed, and ultimately, features with an ICC value exceeding 0.90 were preserved. Radiomics features were extracted using the open-source Pyradiomics package implemented in Python (version 3.7.0) (12), yielding a total of 1,127 radiomics features. All features were normalized, and interobserver consistency analysis was performed. The z-score standardization method was employed for feature normalization. Two parameter sets were employed for discretization to establish binning: binwidth=25, bincount=32.

**Figure 2:**
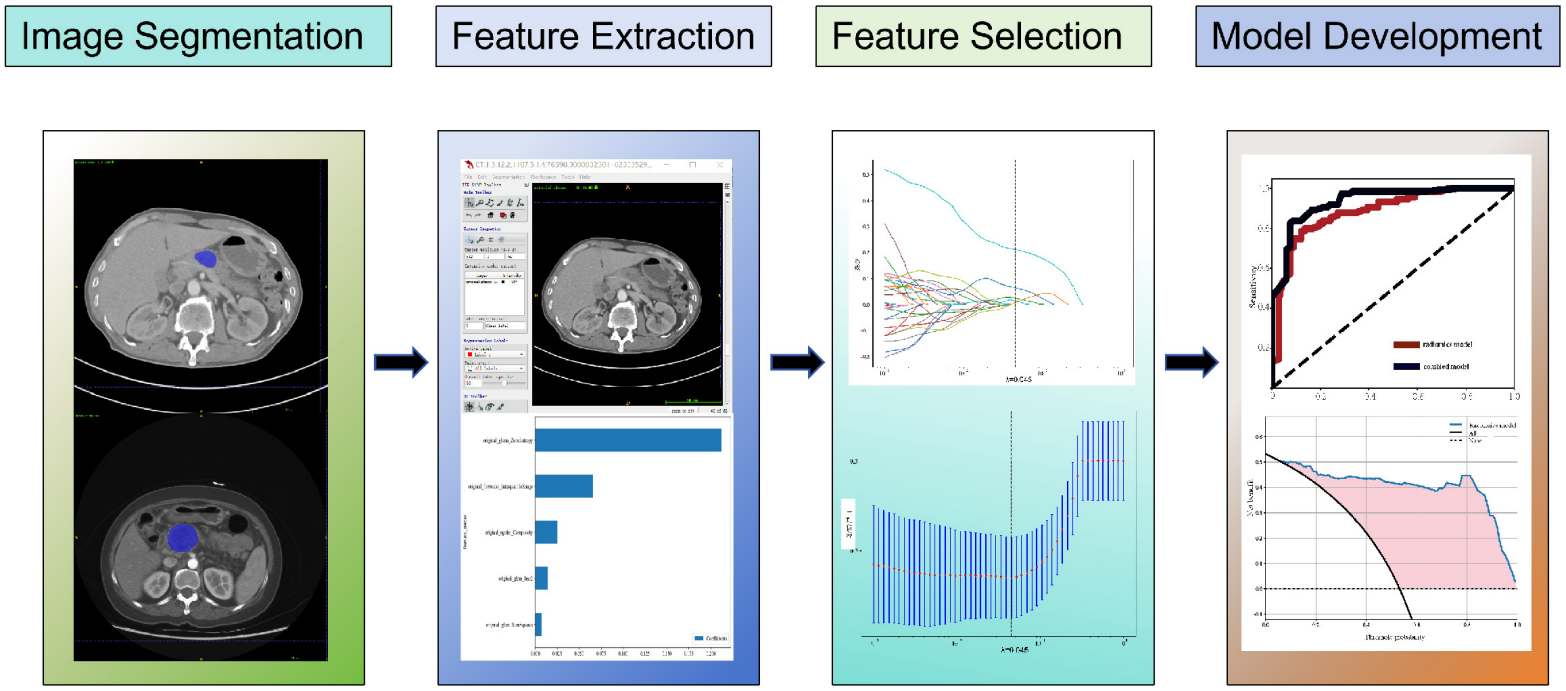
Flowchart of radiomics implementation in this study.

Subsequently, Pearson correlation analysis and the LASSO algorithm were applied for dimensionality reduction and selection of potential discriminative features. To enhance model robustness, 10-fold cross-validation was conducted and repeated 1,000 times, with the optimal penalty parameter (λ) selected based on the minimum criterion. Features with coefficients shrunk to zero were eliminated, and those with higher penalty coefficients were retained as the optimal radiomics features. Radiomics scores were calculated by weighting each selected feature according to its coefficient, and these scores were used for model construction. A RF algorithm was employed to build the radiomics and combined models by aggregating multiple decision trees, thereby improving predictive accuracy and stability (**Figure 2**). The subsequent hyperparameters are: n_estimators=100, max_depth=None, min_samples_split=2, min_samples_leaf=1, and random_state=123.

### Statistical analysis

Statistical analyses were performed using Python and IBM SPSS Statistics (version 26.0). Continuous variables were expressed as mean ± standard deviation and compared using independent-samples t tests. Categorical variables were compared using the chi-square test. Variables with statistical significance in univariate analysis were further entered into multivariate logistic regression analysis to identify independent factors for differentiating IPMN from PDAC. Receiver operating characteristic (ROC) curves with 95% confidence intervals (CIs) were generated, and the DeLong test was used to compare differences between models. Calibration curves and decision curve analysis (DCA) were performed to evaluate model performance and clinical utility. A two-sided *P* value < 0.05 was considered statistically significant.

## Results

### Clinical characteristics and imaging findings

As shown in **Table 1**, the sex distribution was comparable between patients with IPMN and those with PDAC. The mean age at diagnosis was 63.43 ± 9.85 years for the IPMN group and 61.18 ± 10.95 years for the PDAC group. The mean maximum lesion diameter was 15.74 ± 5.12 mm in the IPMN group and 16.34 ± 5.21 mm in the PDAC group (*P* = 0.438). No statistically significant differences were observed between the two groups with respect to gender, age, smoking status, lesion location, maximum diameter, or presence of calcification (all *P* > 0.05). As presented in **Table 2**, multivariate logistic regression analysis demonstrated that single cystic lesion, presence of mural nodules, and elevated CA19–9 levels were independent factors differentiating IPMN from PDAC (all *P* < 0.05), with odds ratios (ORs) of 2.334, 2.690, and 2.358, respectively.

**Table 1:** Clinical characteristics and CT imaging features of patients with IPMN and PDAC.

Characteristics	IPMN(n=82)	PDAC(n=94)	*P* value
Age(years)	63.43 ± 9.85	61.18 ± 10.95	0.16
Gender			0.24
Male	43(52%)	41(44%)	
Female	39(48%)	53(56%)	
Smoking			0.14
No	64(78%)	64(68%)	
Yes	18(22%)	30(32%)	
Lesion location			0.16
Pancreatic head	29(35%)	43(46%)	
Pancreatic body	53(65%)	51(54%)	
Maximum diameter(mm)	15.74 ± 5.12	16.34 ± 5.21	0.44
Single cyst			0.02
Yea	65(79%)	59(63%)	
No	17(21%)	35(37%)	
Mural nodule			0.01
No	56(68%)	44(47%)	
Yes	26(32%)	50(53%)	
Calcification			0.67
No	41(50%)	50(53%)	
Yes	41(50%)	44(47%)	
Lymph nodes			0.03
No	66(80%)	62(66%)	
Yes	16(20%)	32(34%)	
CA19-9			0.01
No	53(65%)	43(46%)	
Yes	29(35%)	51(54%)	

**Table 2:** Multivariate logistic regression analysis of independent predictors differentiating IPMN from PDAC.

Parameters	B value	Standard error	Wald	*P* value	OR (95%CI)
Single cyst	0.85	0.37	5.34	0.02	2.33(1.14-4.79)
Mural nodule	0.99	0.33	8.81	0.03	2.69(1.40-5.17)
CA19-9	0.86	0.33	6.75	0.01	2.36(1.23-4.51)
Lymph nodes	0.71	0.38	3.56	0.06	2.04(0.97-4.28)
Constant	-1.09	0.31	12.46	0.00	0.34

### Feature selection and model construction

**Figure 3:**
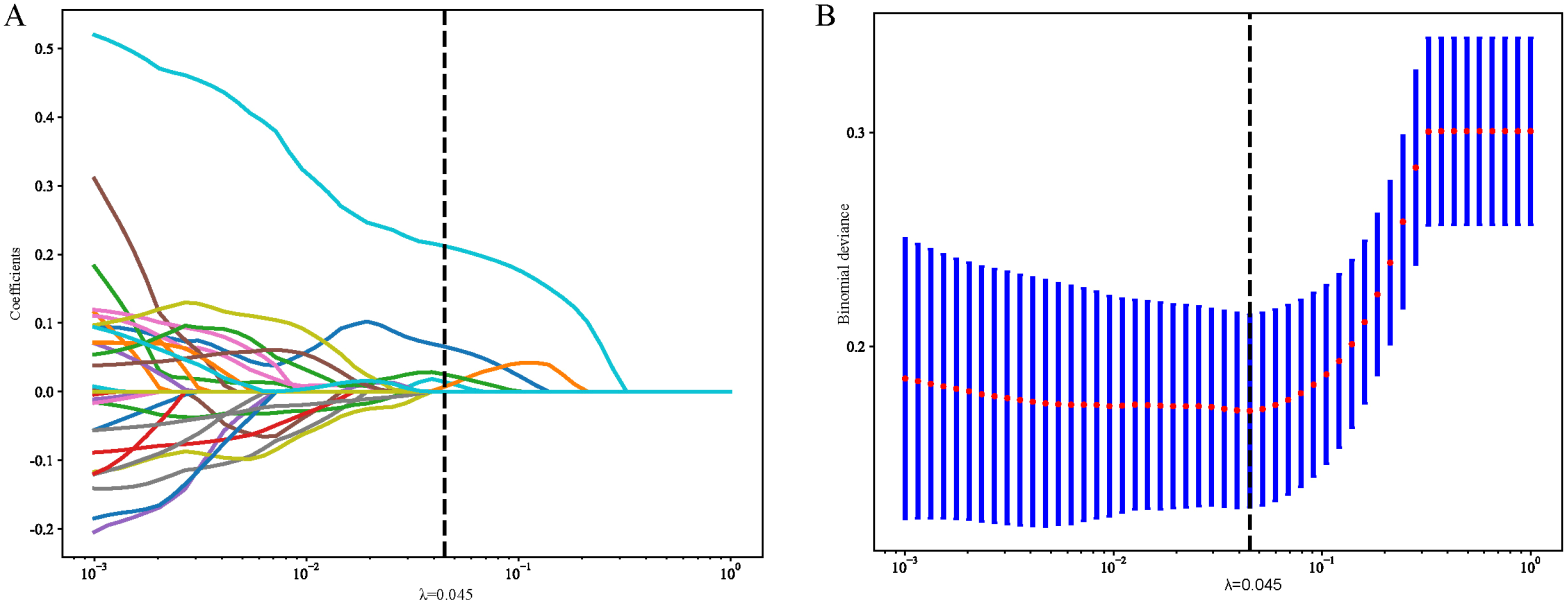
Best radiomics features selected by LASSO regression. **(A)** Radiomics features selected by LASSO regression; **(B)** Optimization of penalty parameter λ determined by 10-fold cross-validation.

**Figure 4:**
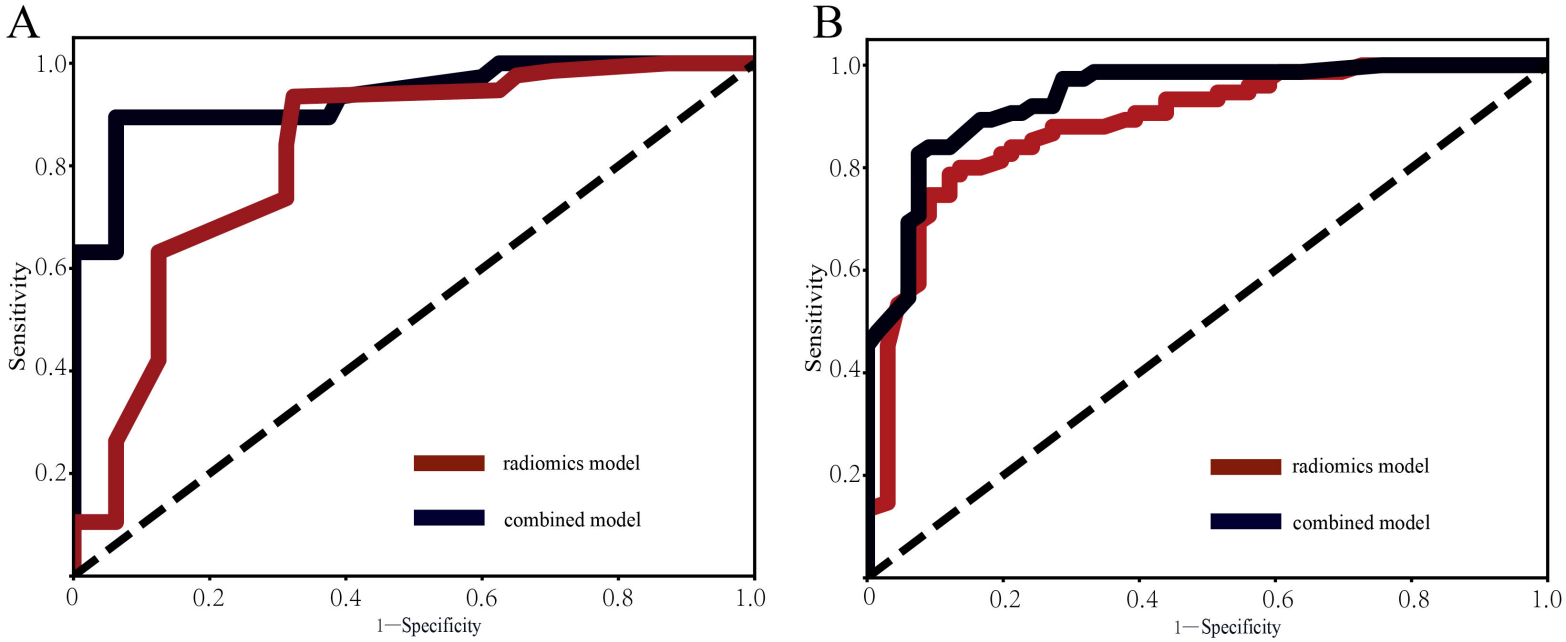
ROC curves of the radiomics model and the combined model. **(A)** Training cohort; **(B)** Test cohort.

**Figure 5:**
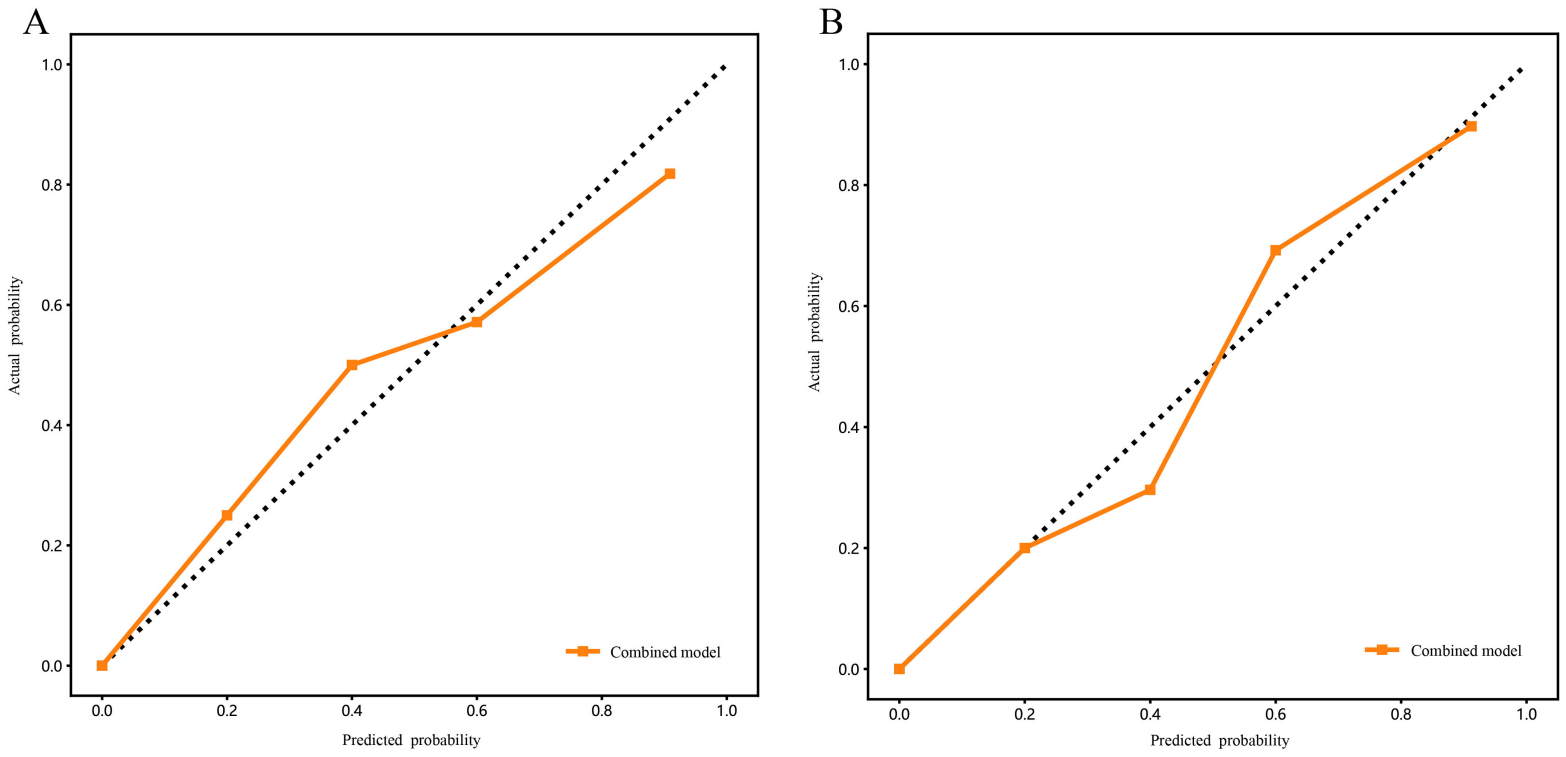
Calibration curves for the combined model. **(A)** Training cohort; **(B)** Test cohort.

**Figure 6:**
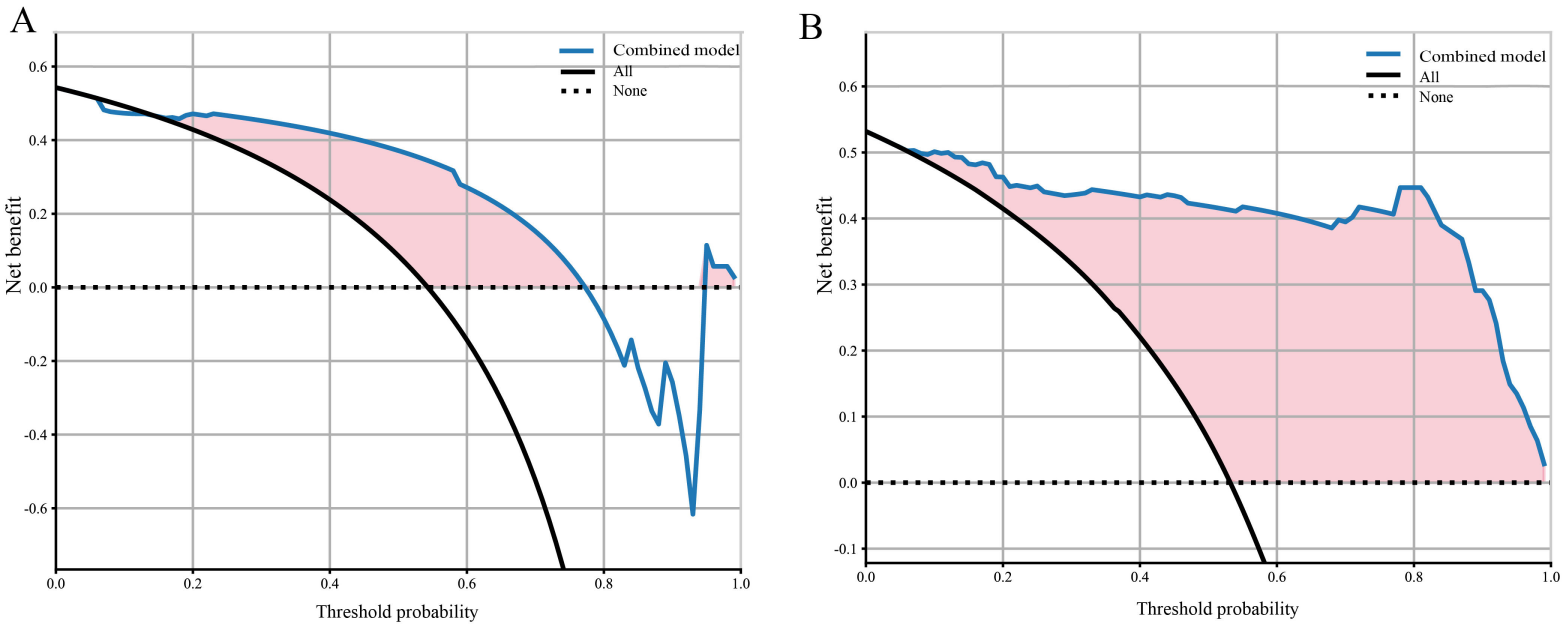
Decision curves for the combined model. **(A)** Training cohort; **(B)** Test cohort.

**Figure 7:**
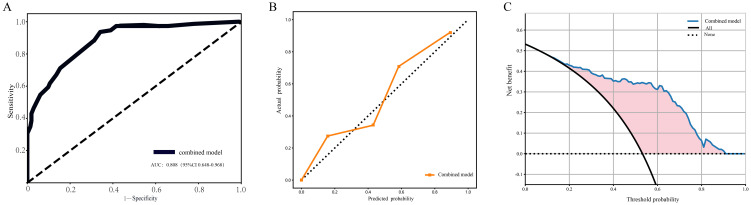
The external validation cohort. **(A)** ROC; **(B)** Calibration curves; **(C)** Decision curves.

A total of 1,127 radiomics features were extracted from the lesion ROIs. Features demonstrating good interobserver reproducibility (ICC > 0.90) and nonzero coefficients were retained for calculation of the radiomics score (**Figure 3**). The radiomics model achieved areas under the ROC curve (AUCs) of 0.885 (95% CI: 0.829-0.941) in the training cohort and 0.827 (95% CI: 0.680-0.975) in the test cohort (*P* = 0.03) (**Table 3**). A combined model incorporating independent clinical predictors demonstrated superior predictive performance, with an AUC of 0.935 (95% CI: 0.896-0.974) in the training cohort and 0.931 (95% CI: 0.846-1.000) in the test cohort (*P* = 0.02) (**Figure 4**). DeLong’s test revealed statistically significant differences between the models in both cohorts (*P* < 0.05). Calibration curves and decision curve analysis indicated good agreement between predicted and observed probabilities for the combined model in both the training and test cohorts (**Figure 5**). Furthermore, DCA demonstrated that the combined model yielded a favorable net clinical benefit (**Figure 6**). The combined model achieved strong performance in the external validation cohort, yielding an AUC of 0.808 (95% CI: 0.684–0.968) and excellent calibration, highlighting its promising utility for clinical decision support (**Figure 7**).

**Table 3:** Performance of different models in differentiating IPMN and PDAC.

Model	AUC (95% CI)	Accuracy	Precision	Sensitivity	Specificity
Training cohort					
Radiomics model	0.885 (0.829 - 0.941)	0.823	0.879	0.773	0.879
Combined model	0.935(0.896 - 0.974)	0.858	0.923	0.800	0.924
Test cohort					
Radiomics model	0.827(0.680 - 0.975)	0.771	0.762	0.842	0.687
Combined model	0.931(0.846 - 1.000)	0.886	0.941	0.842	0.937

## Discussion

As a type of pancreatic cystic neoplasm, IPMN has the potential to progress to invasive PDAC. Although advances in CT technology have markedly improved the detection rate of IPMN, accurately assessing and predicting its malignant potential remains challenging (13). Contrast-enhanced CT imaging offers potential value in differentiating IPMN from PDAC; however, reliable discrimination based solely on visually assessed CT features remains difficult. Radiomics enables the extraction and analysis of large volumes of latent, high-dimensional quantitative features from imaging data, thereby facilitating more precise preoperative differential diagnosis and improving diagnostic accuracy. In the present study, significant differences between IPMN and PDAC were observed with respect to single cystic lesions, presence of mural nodules, and elevated CA19–9 levels. A combined model incorporating radiomics features and clinical variables demonstrated good predictive performance, with AUC values exceeding 0.90 in both the training and test cohorts (0.935 and 0.931, respectively), Additionally, the model also demonstrated strong predictive performance in the external validation cohort.

Previous studies have identified elevated CA19–9 levels, main pancreatic duct diameter of 5-9.9 mm, cyst size ≥40 mm, enhancing mural nodules ≥5 mm, and an annual cyst growth rate of 5 mm as warning indicators of malignant transformation (14). Multivariate logistic regression analyses have further demonstrated that enhancing mural nodules ≥13 mm, obstructive jaundice, and pancreatic duct dilation are independent predictors of malignancy in IPMN (15). Similarly, a study published in 2017 confirmed that the presence of mural nodules and thickened or enhancing cyst walls are associated with higher tumor malignancy grades (16). In a retrospective study by Lee et al., contrast-enhanced CT and MRI showed comparable diagnostic performance and good intermodality agreement in predicting malignancy, with enhancing mural nodules >5 mm showing the strongest association with malignant IPMN (17). In addition, cyst size (≥15 mm), body mass index, and smoking status have been reported as independent risk factors for IPMN (18). Notably, obstructive jaundice, pancreatic duct dilation, and smoking are also recognized risk factors for PDAC. The overlap in risk factors between IPMN and PDAC contributes to the difficulty in achieving accurate differential diagnosis.

Radiomics, as a methodology for extracting quantitative and high-throughput data from medical images, provides features that may reflect underlying pathological and physiological alterations of lesions and potentially reveal tumor phenotypic information. Emerging radiomics features based on quantitative characterization of tumors such as texture, shape, and heterogeneity have shown promise in predicting clinical outcomes in PDAC (19). Cao et al. proposed a novel artificial intelligence model based on non-contrast CT imaging for the early detection of PDAC, thereby potentially transforming current screening strategies. Notably, the performance of this model on non-contrast images was comparable to that of radiologists interpreting contrast-enhanced CT images, suggesting the feasibility of improving preoperative differentiation between PDAC and IPMN (20). Typical features of PDAC include abundant fibrosis, glandular destruction, and low microvascular density. These histopathological characteristics lead to consistent hypoenhancement on arterial and venous phase CT, often accompanied by main pancreatic duct dilation, local atrophy, and biliary obstruction (21).

Previous studies have demonstrated that two-dimensional texture features derived from venous-phase CT images can enable radiologic diagnosis of IPMN with an accuracy of up to 77% (22). During the venous phase, the widespread distribution of the contrast agent leads to a reduced contrast between the tumor and surrounding tissue, causing the image signal to become more homogeneous. Consequently, subtle textural information that reflects the intra-tumoral heterogeneity may be obscured. Leveraging the superior contrast-to-noise ratio in the arterial phase, a joint CT radiomics and machine learning approach can serve as a valuable tool for guiding preoperative therapeutic strategy planning (23, 24). Chen et al. developed a deep learning-based diagnostic approach for PDAC, achieving a sensitivity of 93% and a specificity of 90% (25).

In addition to differentiation from PDAC, IPMN must also be distinguished from other pancreatic lesions, including serous cystadenoma, solid pseudopapillary neoplasm, pancreatic neuroendocrine tumors, pancreatic pseudocysts, and simple mucinous cysts (26, 27). Efforts to elucidate the biological significance of radiomics features are gaining momentum, particularly in exploring their associations with gene expression profiles, microscopic histopathology, and histopathological biomarker expression (23). Whether radiomics features are directly related to visually appreciable characteristics on conventional CT images cannot be determined or inferred from the present study; however, with ongoing advances in science and technology, these questions may be clarified in the future. Concurrently, the emergence of multimodal imaging technologies has been driven by the inherent disparities among different modalities in terms of sensitivity and informational dimension. This trend has been further propelled by hardware advancements, such as hybrid scanners, thereby fostering the development of techniques capable of providing complementary information (28).

This study has several limitations. First, it was a double-center retrospective study with a relatively small sample size, and the conclusions may therefore be subject to selection bias. Second, radiomics feature analysis was performed based on contrast-enhanced CT images; variations in image quality induced by contrast enhancement may have influenced feature extraction and analysis. Additionally, a high-dimensional feature space relative to a small patient cohort predisposes the resulting model to overfitting. Third, the malignant risk of IPMN is associated with its pathological subtypes; however, subgroup analyses were not performed in the present study. With the expansion of sample size in future studies, the relationships between different IPMN subtypes and the risk of progression to PDAC warrant further investigation.

## Conclusions

In conclusion, contrast-enhanced CT-based radiomics can be used for the classification and prediction of IPMN and PDAC. Moreover, integrating radiomics features with clinical variables further improves model performance, providing a noninvasive and personalized assessment for patients and supporting the advancement of precision medicine in clinical practice.

## Data Availability

The original contributions presented in the study are included in the article/supplementary material. Further inquiries can be directed to the corresponding author.
